# FAM3 family genes are associated with prognostic value of human cancer: a pan-cancer analysis

**DOI:** 10.1038/s41598-023-42060-x

**Published:** 2023-09-13

**Authors:** Qing-Tai Dong, Dan-Dan Ma, Qi Gong, Zhen-Yu Lin, Zhong-Hu Li, Jia-Xin Ye, Chun-Hui Qin, Wei-Dong Jin, Jian-Xin Zhang, Zhi-Yong Zhang

**Affiliations:** 1 Department of General Surgery, General Hospital of Central Theater Command, Wuhan, 430070 Hubei China; 2 The First School of Clinical Medicine, Southern Medical University, Guangzhou, Guangdong China

**Keywords:** Cancer, Genetics, Immunology, Biomarkers, Oncology

## Abstract

Family with sequence similarity three member (FAM3) plays a crucial role in the malignant development of various cancers of human. However, there remains doubtful what specific role of FAM3 family genes in pan-cancer. Our study aimed to investigate the role of FAM3 family genes in prognosis, immune subtype, tumor immune microenvironment, stemness score, and anticancer drug sensitivity of pan-cancer. We obtained data from UCSC Xena GDC and CellMiner databases, and used them to study the correlation of the expression, survival, immune subtype, tumor microenvironment, stemness score, and anticancer drug sensitivity between FAM3 family genes with pan-cancer. Furthermore, we investigated the tumor cellular functions and clinical prognostic value FAMC3 in pancreatic cancer (PAAD) using cellular experiments and tissue microarray. Cell Counting Kit-8 (CCK-8), transwell invasion, wound-healing and apoptosis assays were performed to study the effect of FAM3C on SW1990 cells’ proliferation, migration, invasion and apoptosis. Immunohistochemical staining was used to study the relationship between FAM3C expression and clinical characteristics of pancreatic cancer patients. The results revealed that FAM3 family genes are significantly differential expression in tumor and adjacent normal tissues in 7 cancers (CHOL, HNSC, KICH, LUAD, LUSC, READ, and STAD). The expression of FAM3 family genes were negatively related with the RNAss, and robust correlated with immune type, tumor immune microenvironment and drug sensitivity. The expression of FAM3 family genes in pan-cancers were significantly different in immune type C1 (wound healing), C2 (IFN-gamma dominant), C3 (inflammatory), C4 (lymphocyte depleted), C5 (immunologically quiet), and C6 (TGF-beta dominant). Meanwhile, overexpression FAM3C promoted SW1990 cells proliferation, migration, invasion and suppressed SW1990 cells apoptosis. While knockdown of FAM3C triggered opposite results. High FAM3C expression was associated with duodenal invasion, differentiation and liver metastasis. In summary, this study provided a new perspective on the potential therapeutic role of FAM3 family genes in pan-cancer. In particular, FAM3C may play an important role in the occurrence and progression of PAAD.

## Introduction

Cancer has now became the second leading cause of premature death, surpassing infectious diseases^1^. The estimated data on the incidence and mortality of 36 cancers in 185 countries released in 2020 shows that there are 19.3 million new cancer cases, of which 52% has dead^2^. In the future, cancer may surpass cardiovascular disease as the first cause of premature death^1^. In the face of such a serious challenge, many treatment options for cancer have been developed. Targeted therapy of these is a current research hotspot because of its precise treatment sites can reduce drug dose and systemic toxicity compared to conventional therapy^3^. Therefore, it is particularly important to increased research efforts at the genetic level in oncology. Identifying key tumor-related genes can be helpful in understanding the characteristics of cancer^4,5^. Public database contains Multi-omics, large sample, immune subtype and drug sensitivity data, which make it enables polygenic pan-cancer studies^6^.

The tumor microenvironment (TME) comprises stromal cells, fibroblasts, endothelial cells, immune cells and noncellular components^7,8^. It plays an important role in cancer development, as it can provide a suitable place for tumor cells to survive^9,10^. It has been shown that combining existing therapies with modulate TME can improve the effect of cancer therapy^11,12^. Thorsson et al. identify six stable and reproducible immune subtypes which are associated with prognostic, genetic and immune modulatory alterations and play a key role in predicting disease prognosis^13^. Their research shows that both across and within immune subtypes are involved in tumor-immune cell interactions. Therefore, the study for TME and immune subtype have great implications for the treatment of cancer.

The family with sequence similarity 3 (FAM3) has 4 members (FAM3A, FAM3B, FAM3C and FAM3D) belongs to cytokine-like gene family^14,15^. Previous studies have shown that FAM3 has important functions in diabetes, Alzheimer disease and cancer^16–19^. In particular, FAM3A is highly expressed in almost all tissues, which overexpression can elevate intracellular and extracellular ATP contents and facilitate PDX1 expression and insulin secretion^14,20^. FAM3B is originally found in pancreas and involved in glucose metabolism and lipogenesis^14,21^. Interestingly, FAM3B plays an important role in cancer initiation and progression. Up-regulated FAM3B can promote invasion and metastasis of human colon cancer, esophageal squamous cell carcinoma and prostate cancer cells and is correlated with bad prognosis of the patients^22–24^. Elevated FAM3C is strongly associated with poor prognosis in most human cancers, such as liver cancer, colorectal cancer, gastric cancer, breast cancer, esophageal squamous cell carcinoma and oral squamous cell carcinoma^25^. Whereas, the relationship between FAM3C and pancreatic cancer (PAAD) remains elusive. FAM3D is a gut-secreted protein, which levels are regulated by nutritional status^26^. Meanwhile, FAM3D is essential in colon homeostasis and protection against inflammation associated cancer^27^. However, the biological functions of each member of the FAM3 family genes in pan-cancer were not fully understood, particularly with respect to the TME and immune subtype.

In our study, we first analysed the correlation of expression and survival between FAM3 family genes and 33 human cancers. Meanwhile, the relevance of the FAM3 family genes, immune subtype, TME, stemness score, and drug sensitivity of pan-cancer were explored. Furthermore, we evaluated the prognostic significance of FAM3C in pancreatic cancer and validated the role of FAM3C in PAAD by in vitro cellular assays. This study may pave a novel road for exploring the clinical implications of FAM3 family genes in pan-cancer.

## Materials and methods

### Acquisition of datasets for bioinformatics analysis

Transcription expression (RNA-sep—FPKM), phenotype and survival data of 33 human malignant tumors were download from GDC TCGA of UCSC Xena (https://xenabrowser.net/datapages/). They were acute myeloid leukemia (LAML), adrenocortical cancer (ACC), bile duct cancer (CHOL), bladder cancer (BLCA), breast cancer (BRCA), cervical cancer (CESC), colon cancer (COAD), endometrioid cancer (UCEC), esophageal cancer (ESCA), glioblastoma (GBM), head and neck cancer (HNSC), kidney chromophobe (KICH), kidney clear cell carcinoma (KIRC), kidney papillary cell carcinoma (KIRP), large B-cell lymphoma (DLBC), liver cancer (LIHC), lower grade glioma (LGG), lung adenocarcinoma (LUAD), lung squamous cell carcinoma (LUSC), melanoma (SKCM), mesothelioma (MESO), ocular melanomas (UVM), ovarian cancer (OV), pancreatic cancer (PAAD), pheochromocytoma and paraganglioma (PCPG), prostate cancer (PRAD), rectal cancer (READ), sarcoma (SARC), stomach cancer (STAD), testicular cancer (TGCT), thymoma (THYM), thyroid cancer (THCA), and uterine carcinosarcoma (UCS). Immune subtype, RNA based (RNA-exp) and DNA methylation based (DNA-meth) stemness scores data were download from TCGA Pan-Cancer of UCSC Xena. DTP NCI-60 average z scores and RNA-exp composite expression data were download from CellMiner (https://discover.nci.nih.gov/cellminer/loadDownload.do).

### Gene expression analysis and survival analysis

We extracted the transcription expression data of FAM3 family genes in 33 tumor and adjacent normal tissues using the “limma” R package. The extraction condition was set to take the mean value if a gene occupied multiple rows, resulting in 11,057 samples. We analysed FAM3 family genes expression between tumor and adjacent normal tissues of 24 cancers using the wilcox test, because of there were no corresponding normal samples for the remaining 9 cancers (LAML, ACC, DLBC, LGG, MESO, UVM, OV, TGCT, UCS). Wilcoxon test was performed in gene differential expression analysis. “Survival” and “survminer” R package were used to analyse overall survival of pan-cancer association with FAM3 family genes. Kaplan–Meier and Cox methods were performed in survival analysis.

### FAM3 family genes correlation analysis

Based on the analysis of gene expression in pan-cancer, in order to identify the correlation relationship between FAM3 family genes by using “corrplot” R package. The association coefficient was bounded by 0 and ranges from − 1 to 1. It meant there was a positive correlation between FAM3 family genes when the association coefficient was greater than 0, and when the association coefficient was closer to 1, it indicated a stronger positive correlation. Conversely, it meant there was a negative correlation between the FAM3 family genes when the correlation coefficient was less than 0, and when the correlation coefficient was closer to − 1, it indicateda stronger negative correlation.

### Immune subtype analysis

In this study, immune subtype data included a total of 9126 samples of six immune subtypes, namely wound healing (immune C1), IFN-gamma dominant (immune C2), inflammatory (immune C3), lymphocyte depleted (immune C4), immunologically quiet (immune C5), and TGF-beta dominant (immune C6). We analysed the correlation of pan-cancer and single tumour immune subtypes association with FAM3 family genes expression using “limma” and “reshape2” R package. Kruskal–Wallis test was performed in immune subtype analysis.

### Correlation analysis of the tumor microenvironment and tumor stem cell score

The immune score, stromal score, and estimate score of pan-cancer were calculated from the transcription expression data by using “estimate” R package. We extracted RNAss and DNAss information of pan-cancer from RNA-exp and DNA-meth stemness scores data using “limma” R package. Then, we combined FAM3 family genes transcription expression data with immune score, stromal score, estimate score, RNAss (based on RNA expression), and DNAss (based on DNA methylation) for correlation analysis using “corrplot” R package. Spearman correlation test was performed in correlation analysis.

### Drug sensitivity analysis

CellMiner, compiled by the US National Cancer Institute, is a freely available tool that organizes and stores the raw and normalized data for multiple types of molecular characterizations, such as the DNA, RNA, protein, and pharmacological levels^28,29^. The higher value of DTP NCI-60 average z scores, the more drug’s anticancer activity. We filtered out drug sensitivity data from DTP NCI-60 average z scores data by the conditions for which there were clinical trial and FDA approved in the column headed “FDA status”. Meanwhile, we extracted the same sample of gene expression data from RNA-exp composite expression data. Next, we combined FAM3 family genes expression with drug sensitivity data to analyse drug sensitivity using pearson correlation test.

### Differential expression analysis, clinical characteristic analysis, and Gene Set Enrichment Analysis (GSEA) of FAM3C in PAAD

In our study, we tried further to explore the prognostic significance of FAM3C in PAAD. However, there were only 4 normal samples in PAAD transcription expression data of TCGA. To reduce sampling error, we performed FAM3C expression analysis of PAAD using GEPIA2 (http://gepia2.cancer-pku.cn/#index) which contain TCGA and GTEx data. Wilcoxon test, Kruskal–Wallis test, and Cox regression were used to evaluate the relationships between clinical characteristics and FAM3C expression. GSEA was performed, and gene expression data was divided into two groups based on the median FAM3C expression level: high and low.

### Tissue samples and immunohistochemistry (IHC) analysis

Tissue microarray (TMA, n = 133, Cat No: T20-1179-12) was obtained from Shanghai Outdo Biotech Co., LTD, including 115 PAAD tissues which contain detailed clinical characteristics information (As described in Table 1) and 18 adjacent normal tissues. EnVision System (Dako Diagnostics, Switzerland) was performed to stain the TMA specimens. The TMA slides were incubated overnight at 4℃ with rabbit polyclonal antibody against FAM3C (Cat No: 14247-1-AP, 1:50, Proteintech, USA). After washing with phosphate buffered saline (PBS), substrate-chromogen and peroxidase labelled polymer were applied to visualize the protein staining. Then, we used the CaseViewer 2.4 software (3DHISTECH Ltd, Hungary) to analyzed the TMA slides. Two investigators with pathological backgrounds used the visual immunoreactive score (IRS) to evaluate all TMA sliders. The score criteria of staining intensity (SI) as follows^30,31^: none = 0, weak = 1, moderate = 2, and strong = 3. The percentage of positive cells: negative = 0, < 10% positive cells = 1, 10% ~ 50% positive cells = 2, 51% ~ 80% positive cells = 3, > 80% positive cells = 4. The final IRS was calculated by SI × percentage of positive cells. We divided the samples into low (IRS ≤ 4) or high expression (IRS > 4).

**Table 1:** Relationship between the expression of FAM3C in pancreatic carcinoma tissues and the clinical characteristics of pancreatic carcinoma patients (n = 115).

Parameters	TMA			
	High, n (%)	Low, n (%)	*χ*^2^	*P* value
Gender				
Male	51 (58.6)	36 (41.4)	2.125	0.145
Female	12 (42.9)	16 (57.1)		
Age, years				
≤ 60	39 (54.2)	33 (45.8)	0.029	0.864
> 60	24 (55.8)	19 (44.2)		
Tumor location				
Head	50 (52.6)	45 (47.4)	1.020	0.312
Body or tail	13 (65.0)	7 (35.0)		
Tumor size, cm				
≤ 2	17 (42.5)	23 (57.5)	3.735	0.053
> 2	46 (61.3)	29 (38.7)		
Neural invasion				
Yes	30 (63.8)	17 (36.2)	2.626	0.105
No	33 (48.5)	35 (51.5)		
Duodenal invasion				
Yes	13 (81.3)	3 (18.8)	5.256	0.022
No	50 (50.5)	49 (49.5)		
Differentiation				
Low	20 (74.1)	7 (25.9)	7.317	0.026
Median	39 (52.0)	36 (36.0)		
High	4 (30.8)	9 (69.2)		
Lymphatic invasion				
Yes	26 (65.0)	14 (35.0)	2.585	0.108
No	37 (49.3)	38 (50.7)		
Vascular invasion				
Yes	20 (64.5)	11 (35.5)	1.623	0.203
No	43 (51.2)	41 (48.8)		
Liver metastasis				
Yes	12 (80.0)	3 (20.0)	4.428	0.035
No	51 (51.0)	49 (49.0)		
TNM stage (UICC)				
I or IIA	32 (48.5)	34 (51.5)	2.480	0.115
IIB or III, IV	31 (63.3)	18 (36.7)		

*UICC* Union for International Cancer Control. Bold indicates statistically significant at *P* value < 0.05.

### Cell culture and transfection of PAAD

The human PAAD cell line SW1990 was purchased from the Type Culture Collection of the Chinese Academy of Sciences. Cells were cultured in Dulbecco’s modified eagle medium (DMEM) (Gibco, USA) supplemented with 10% foetal bovine serum (FBS) (Gibco, USA), and 1% penicillin–streptomycin solution (Beyotime, China). The cultured cells were incubated at 37 °C with 5% CO_2_. For cell transfection, six-well plates were used. The synthesized pcDNA3.1-FAM3C (FAM3C) and the empty plasmid pcDNA3.1 (Control) were purchased from the MiaoLing Plasmid Sharing Platform. The sequence of the siRNA targeting FAM3C (siRNA-FAM3C) was synthesized (Genepharma, China) as follows: 5′-ACUUUUCAUUAAUAUGCUCCG-3′. Meanwhile, as a control treatment, scrambled siRNA (siRNA-Control) was created. The transfection into cells of SW1990 was performed by using the Lipofectamine 2000 (Thermo, USA). The transfection efficiency was determined by western blotting at 48 h post-transfection.

### Cell proliferation, wound-healing, transwell invasion, and apoptosis assay of PAAD

For cell proliferation assay, we applied Cell Counting kit-8 (CCK-8) (Beyotime, China) assay. After 24 and 48 h of transfection, the cells were plated in 96-well plates at a concentration of 5 × 10^3^ cells per well in 100 µl complete growth medium. Next, each well received 10 µl CCK-8 reagent, and cells were incubated for another 2 h. A microplate reader (Thermo, USA) was used to measure the optical density (OD) of each well at 450 nm. For Wound-healing assay, we seeded Transfected cells (5 × 10^5^ cells/well) into 6-well plates with a fresh medium containing 10% FBS, and incubated until > 90% confluence was reached. To create a linear scratch wound, the cell monolayer was scratched with a 10 µl sterile plastic pipette tip. The floating cells were washed three times with phosphate buffer saline (PBS) and the medium was replace with serum-free medium with 4 μg/ml mitomycin. Eventually, an inverted light microscope (magnification, × 200; Olympus Corporation) was used to capture scratch-wound images at 0 and 48 h. For transwell invasion assay, we added cell dilution (100 µl, 5 × 10^4^ cells in serum-free DMEM medium) to the upper chamber, which contained an 8 μm polycarbonate filter (Millipore, USA) pre-coated with 100 µl diluted Matrigel (BD, USA). The lower chamber was filled with DMEM medium with 10% FBS. After incubation at 37 °C with 5% CO_2_ for 24 h, the non-migrated cells at the upper surface were removed. The invading cells on the lower surface were fixed for 15 min in 4% paraformaldehyde and stained for 20 min in 0.1% crystal violet. The images of cell counts were captured under an inverted microscope. Eventually, we further detected cell apoptosis using Annexin V-fluorescein isothiocyanate (FITC)/propidium iodide (PI) detection kit (BD, USA) following the manufacturer's protocol. Transfected cells (1 × 10^6^ cells/well) were seeded into 6-well plates. Following incubation at 37 °C with 5% CO_2_ for 48 h. Cells were collected and washed twice with PBS and resuspended in 100 µl binding buffer (Thermo, USA). Then, Annexin V-FITC (5 µl) and PI (5 µl) were added and continuously incubated for 30 min in darkness at room temperature. Finally, a FACS Calibur flow cytometer (BD, USA) was used to analysis the percentage of apoptotic cells.

### Statistical analysis

The statistics from TCGA were analyzed by R (v.4.1.1). Gene set enrichment analysis was performed using GSEA (v.4.1.0) software, and multiple testing correction applied. The statistical analyses of cellular functions were performed using GraphPad Prism Software 8.0. Descriptive statistics were presented as the means ± SD. *P* < 0.05 was considered as statistically significant.

## Results

### The expression levels of FAM3 family genes in 24 cancers

**Figure 1:**
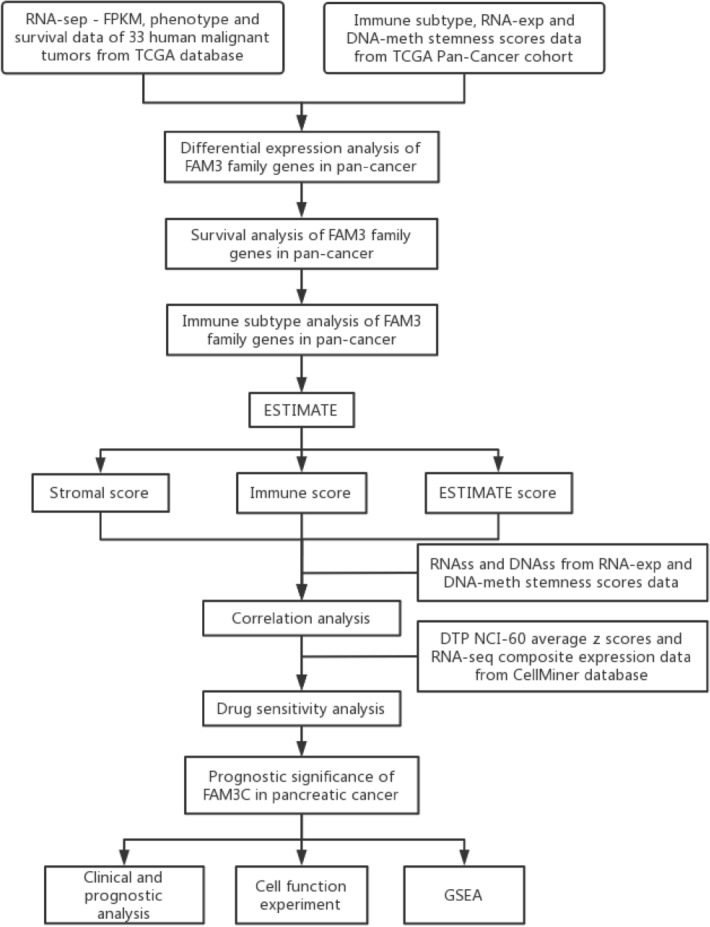
Flowchart of this study.

**Figure 2:**
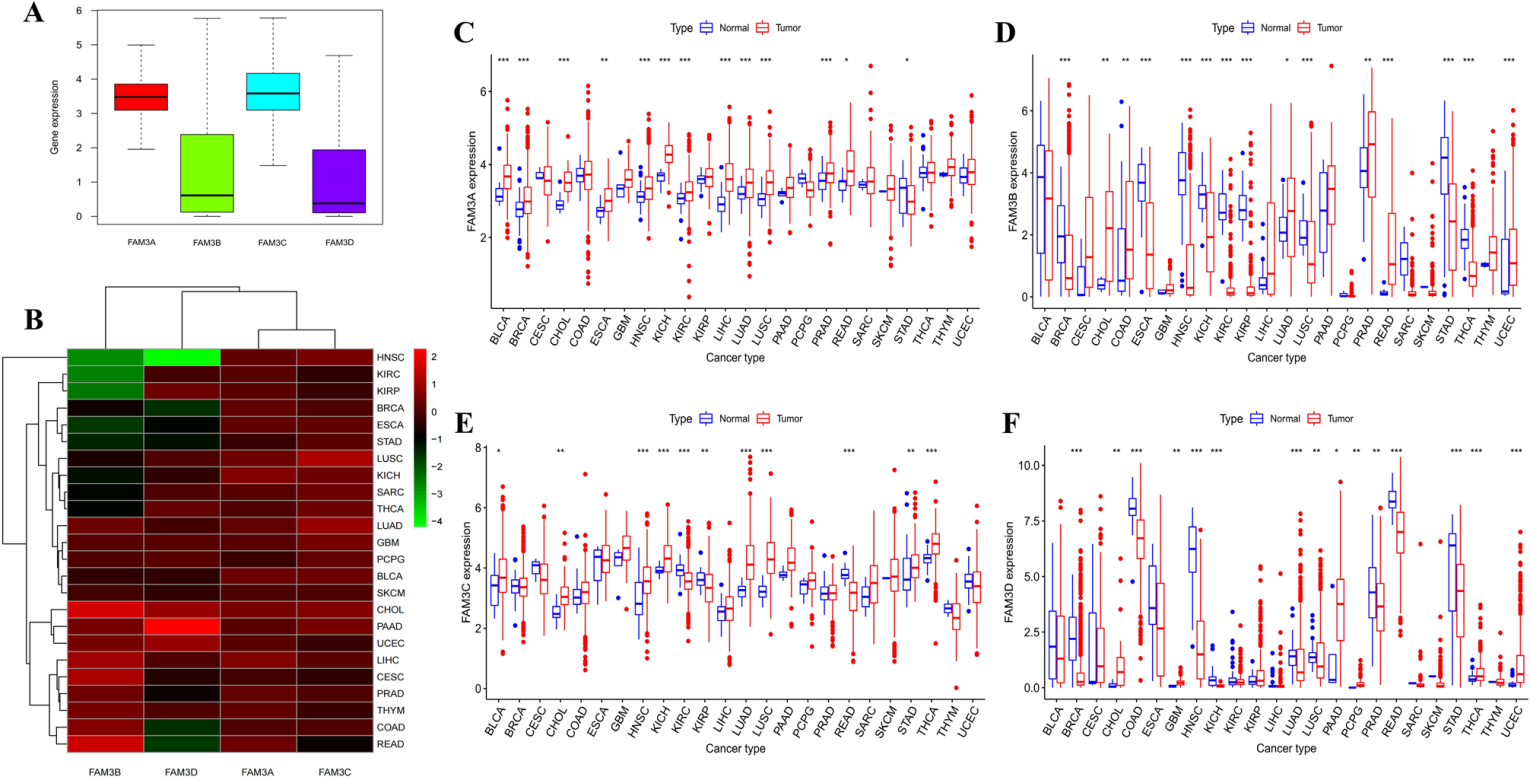
The expression of FAM3 family genes in pan-cancer. (**A**) Box plot of FAM3 family genes expression in 33 cancer tissues. (**B**) Heatmap of FAM3 family genes expression in 24 cancer tissues. Red boxes indicate high expression of the gene in this tumor, and conversely green boxes indicate low expression. (**C–F**) Differential expression analysis in 24 cancers of FAM3A (**C**), FAM3B (**D**), FAM3C (**E**), FAM3D(**F**). *P < 0.05; **P < 0.01; ***P < 0.001.

The flow diagram of the whole study is shown in Fig. 1. We obtained the results of FAM3 family gene expression in 24 cancers as shown in Fig. 2. Figure 2A showed that the expression of FAM3A and FAM3C were higher in 33 cancer tissues relative to FAM3B and FAM3D, with the highest expression of FAM3C. As shown in Fig. 2B–F, we found that FAM3 family genes were significantly differential expression in tumor and adjacent normal tissues in 7 cancers (CHOL, HNSC, KICH, LUAD, LUSC, READ, and STAD) (*P* < 0.05). From the heatmap of the log (fold change (FC)) of FAM3 family genes in 24 cancers, we can see that FAM3A and FAM3C showed a tendency to be highly expressed in the tumor tissues (as Fig. 2B). Some cancer types (PAAD, CECS, PCPG, SARC, SKCM, and THYM) in the TCGA database have fewer than 5 normal tissues, which may bias the statistical analysis when performed, leading to inaccurate results. Therefore, whether there was differential expression of FAM3 family genes in these cancer types required a larger sample of normal tissues for analysis.

### The prognosis and FAM3 family genes expression in pan-cancer

**Figure 3:**
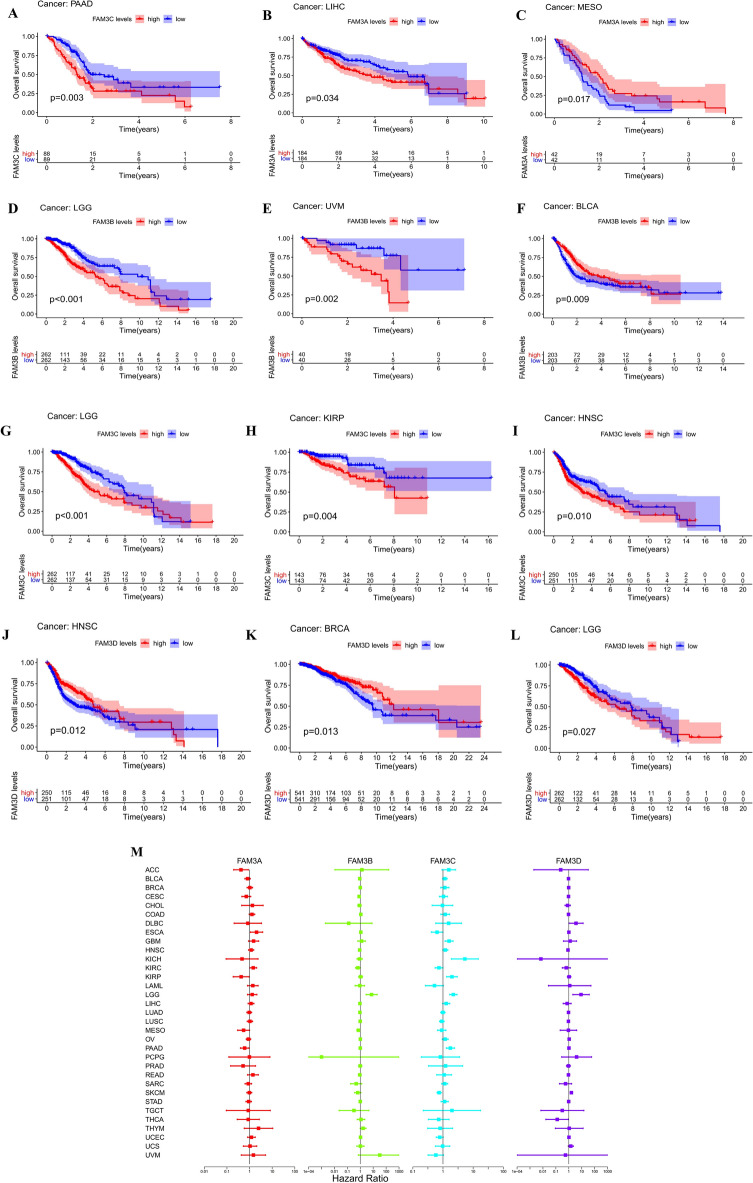
Survival analysis of FAM3 family genes in pan-cancer. (**A**) Survival curve of FAM3C in PAAD (*P* = 0.003). (**B**,**C**) Survival curve of FAM3A in LIHC (*P* = 0.034) (**B**), MESO (*P* = 0.017) (**C**). (**D–F**) Survival curve of FAM3B in LGG (*P* < 0.001) (**D**), UVM (*P* = 0.002) (**E**), BLCA (*P* = 0.009) (**F**). (**G–I**) Survival curve of FAM3C in LGG (*P* < 0.001) (**G**), KIRP (*P* = 0.004) (**H**), HNSC (*P* = 0.010) (**I**). (**J–L**) Survival curve of FAM3D in HNSC (*P* = 0.012) (**J**), BRCA (*P* = 0.013) (**K**), LGG (*P* = 0.027) (**L**). (**M**) Forest map of hazard ratio of FAM3 family genes in pan-cancer.

The results of the prognostic impact of the FAM3 family genes on 33 cancer patients were shown in Fig. 3 and Supplementary Fig. 1, with a total of 20 statistically significant results. As shown in Fig. 3B,C, The FAM3A high expression group had a poor prognosis compared to the low in LIHC patients (*P* < 0.05). However, The FAM3A low expression group had a poor prognosis compared to the high in MESO patients (*P* < 0.05). The FAM3B high expression group had a poor prognosis compared to the low in LGG and UVM patients (*P* < 0.01) (Fig. 3D,E). The FAM3B low expression group had a poor prognosis compared to the high in BLCA patients (*P* < 0.05) (Fig. 3F). As for FAM3C, its high expression association with poor prognosis compared to the low in PAAD, LGG, HNSC, and KIRP patients (*P* < 0.05) (Fig. 3A,G–I)). The FAM3C low expression group had a poor prognosis compared to the high in SKCM and ESCA patients (*P* < 0.05) (Supplementary Fig. 1). We can see that the FAM3D high expression group had a poor prognosis compared to the low in LGG patients (*P* < 0.05) (Fig. 3L). The FAM3D low expression group had a poor prognosis compared to the high in HNSC, and BRCA patients (*P* < 0.05) (Fig. 3J,K). Figure 3M showed that FAM3A was a high-risk factor for ESCA and KIRC patients. FAM3B was a high-risk factor for LGG patients. FAM3C was a high-risk factor for PAAD, LGG, KIRP, KICH, and GBM patients. FAM3D was a high-risk factor for LGG, SKCM, and DBLC patients.

### Correlation analysis of FAM3 family genes and immune subtype of pan-cancer and single tumor

**Figure 4:**
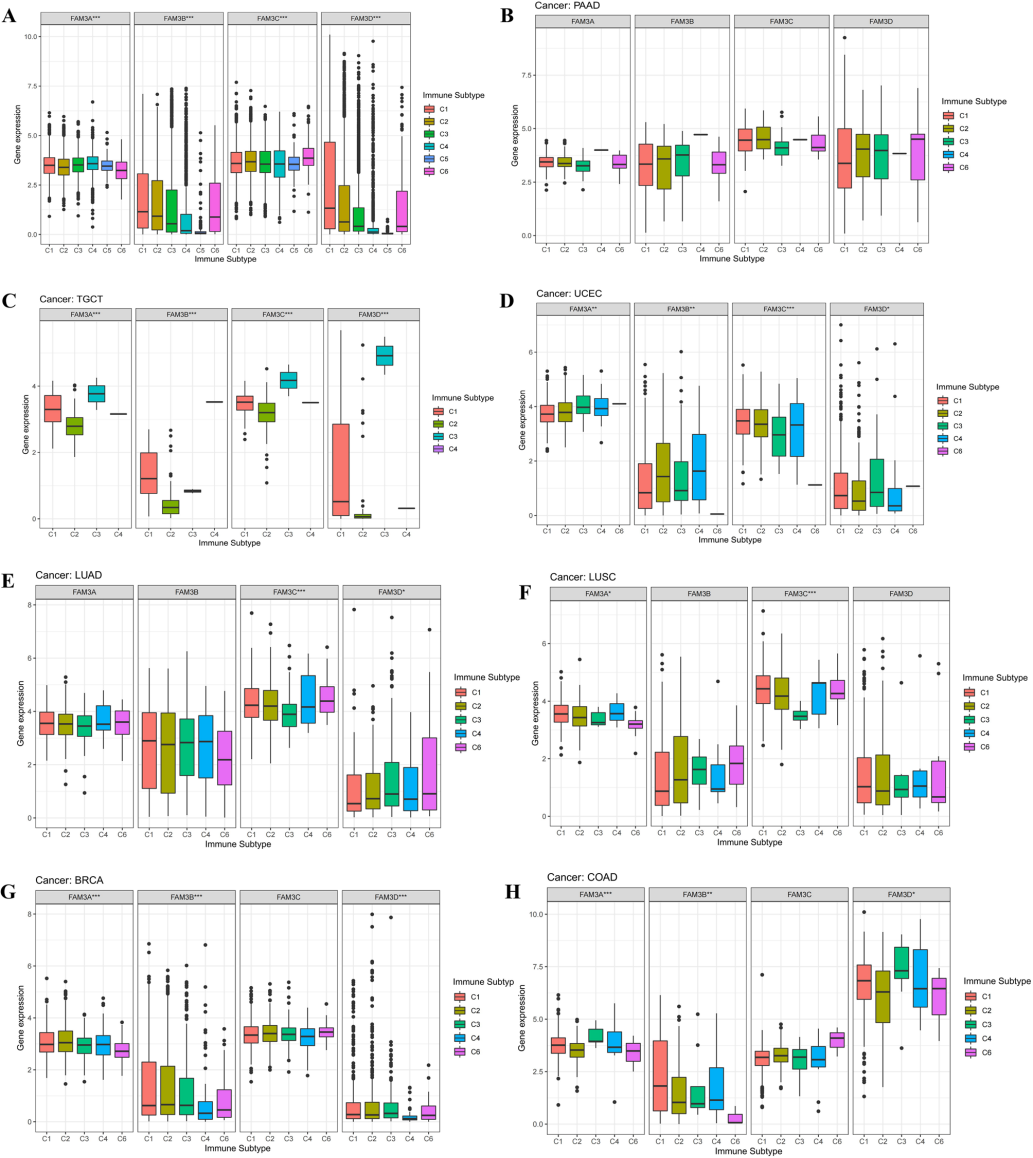
Immune subtype analysis of FAM3 family genes in pan-cancer. (**A**) Box plot of FAM3 family genes differential expression in immune subtype of pan-cancer. (**B–H**) Box plot of FAM3 family genes differential expression in immune subtype of PAAD (**B**), TGCT (**C**), UCEC (**D**), LUAD (**E**), LUSC (**F**), BRCA (**G**), COAD (**H**). *P < 0.05; **P < 0.01; ***P < 0.001.

In the results of 33 cancers immune subtype analysis, we found that the expression of FAM3 family genes in pan-cancers were significantly different in immune type C1, C2, C3, C4, C5, and C6 (*P* < 0.001) (Fig. 4A). The results of single tumor immune subtype analysis were showed in Fig. 4B–H. FAM3 family genes expression in PAAD were not significantly different in immune type C1, C2, C3, C4, and C6 (*P* > 0.05) (Fig. 4B). The expression of FAM3 family genes in TCTG were significantly different in immune type C1, C2, C3, and C4 (*P* < 0.001) (Fig. 4C). The expression of FAM3 family genes in UCEC were significantly different in immune type C1, C2, C3, C4, and C6 (*P* < 0.01) (Fig. 4D). FAM3C expression in LUAD and LUSC were significantly different in immune type C1, C2, C3, C4, and C6 (*P* < 0.001) (Fig. 4E,F). However, the expression of FAM3C in BRCA and COAD were not significantly different in immune type C1, C2, C3, C4, and C6 (*P* > 0.05) (Fig. 4G,H).

### Correlation analysis of FAM3 family genes and tumor microenvironment and stemness scores in pan-cancer

**Figure 5:**
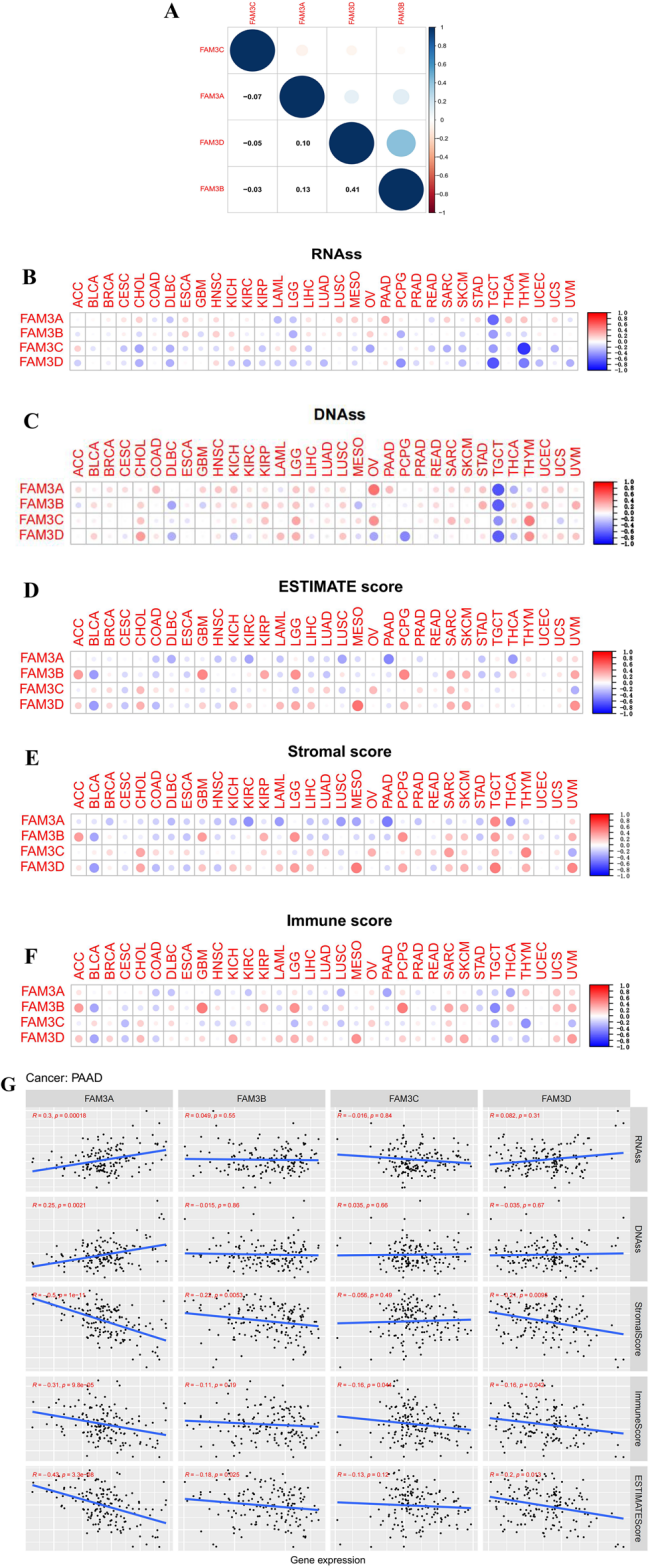
Correlation analysis of FAM3 family genes expression and TME and stemness scores. (**A**) Mutual correlation analysis of FAM3 family genes in pan-cancer. Blue dots indicate positive correlation, while the opposite red dots indicate negative correlation. (**B–F**) The correlation relationship between FAM3 family genes expression and RNAss (**B**), DNAss (**C**), estimate score (**D**), stromal score (**E**), immune score (**F**). Red dots indicate positive correlation, while blue dots indicate negative correlation, and larger dots indicate stronger correlation and vice versa. (**G**) Correlation analysis between FAM3 family genes expression and TME and stemness scores in single tumor PAAD.

As we can see, Fig. 5A showed that FAM3C expression was negatively correlated with FAM3A, FAM3B, and FAM3D expression in pan-cancer (Cor = − 0.07, − 0.05, and − 0.03). FAM3A expression was positively correlated with FAM3D, and FAM3B expression in pan-cancer (Cor = 0.10, and 0.13). FAM3B expression was positively correlated with FAM3D expression in pan-cancer (Cor = 0.41). From Fig. 5B,C we can find that most of FAM3 family genes expression were negatively correlated with the RNAss and DNAss of pan-cancer. The results of TME include stroma, immune cell and total score (estimate score = stromal score + immune score). As Fig. 5D–F. FAM3A was negatively correlated with stromal score, immune score and estimate score in most of pan-cancer, which indicated that the higher expression of FAM3A, lower TME score, meant the higher content of tumor cells. On the one hand, the results above showed that FAM3C expression was highest in pan-cancer. On the other hand, high FAM3C expression was strongly associated with poorer prognosis in PAAD, LGG, HNSC and KIRP, and when we analysed the differences in the expression of FAM3C in PAAD, LGG, HNSC and KIRP according to conjunction with more data from the TCGA and GTEx databases, we found that only FAM3C expression in PAAD was significantly higher than normal tissues. In order to verify the reliability of the results of this study, we analysed the relationship between FAM3C and PAAD separately in the next study by combining relevant clinical data and cellular experiments. Therefore, we further analyzed the relationship between FAM3 family genes and TME and tumor stem cell score in pancreatic cancer as shown in Fig. 5G. FAM3A expression was significantly positively correlated with the RNAss and DNAss of PAAD (*P* < 0.01). Meanwhile, FAM3A expression was significantly negatively correlated with stromal score, immune score, and estimate score of PAAD (*P* < 0.001). FAM3B expression was significantly negatively correlated with stromal score and estimate score of PAAD (*P* < 0.05). FAM3D expression was significantly negatively correlated with stromal score, immune score, and estimate score of PAAD (*P* < 0.05).

### Correlation analysis between FAM3 family genes expression and drug sensitivity

**Figure 6:**
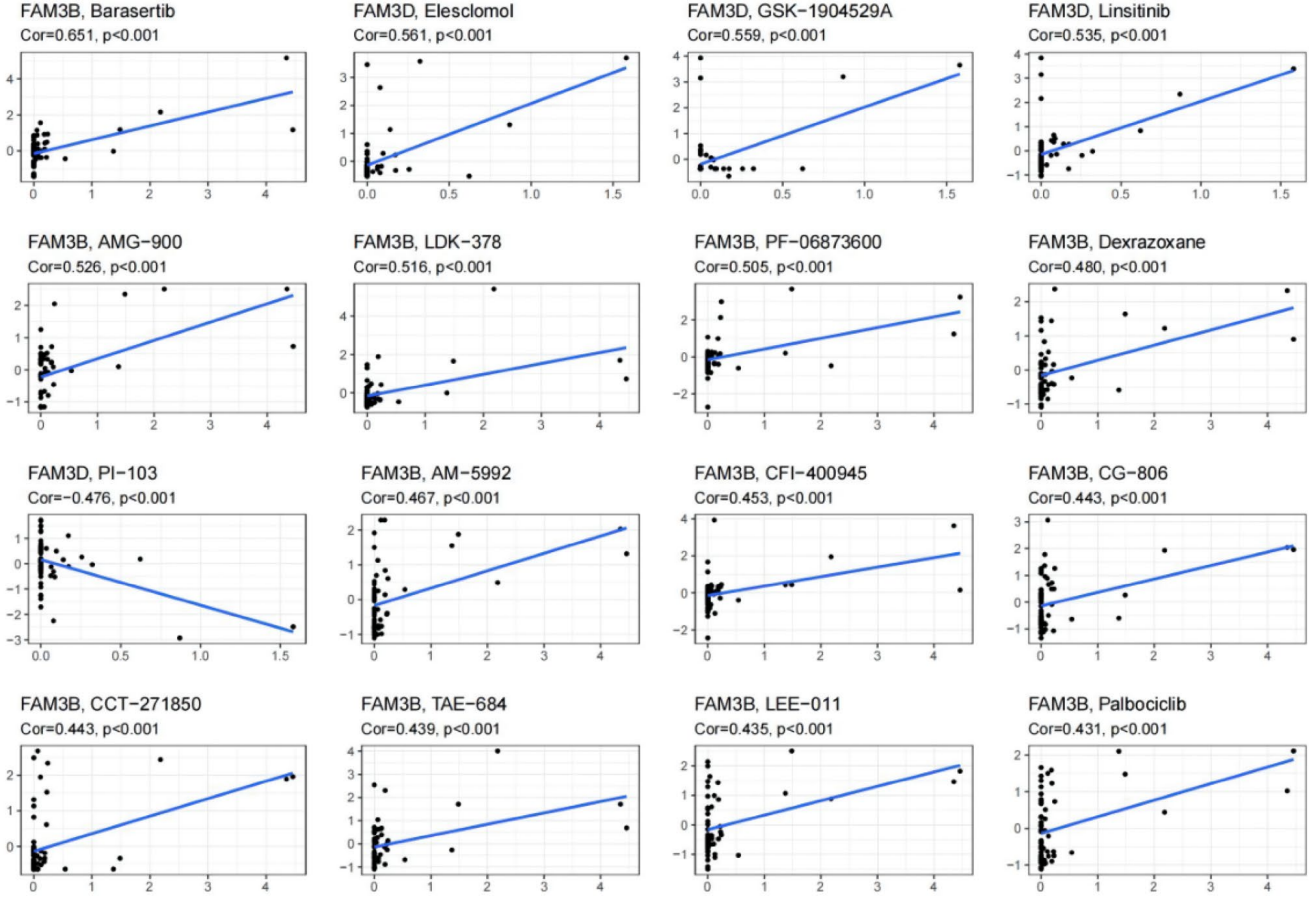
Correlation analysis between FAM3 family genes expression and drug sensitivity in CellMiner. The top 16 analysis results with the smallest *P* values based on correlation analysis.

From 792 drugs sensitivity analysis results. In order of absolute value of Cor values, and *P* < 0.05 was considered significant. We obtained 247 significant results (Supplementary Table 1). Each member expression degree of FAM3 family genes can alter the sensitivity of tumour cells to certain drugs. FAM3B and barasertib have the highest correlation (Cor = 0.651, *P* < 0.001). FAM3B influenced the most drug types (up to 89), and its expression was positively correlated with sensitivity to most drug types (up to 78), implying that patients with high FAM3B expression may be better treated with anticancer drugs. We selected the top 16 analysis results with the smallest p-values based on correlation analysis, as shown in Fig. 6. The higher FAM3B expression, the stronger drug sensitivity of AMG-900, LDK-378, PF-06873600, dexrazoxane, AM-5992, CFI-400945, CG-806, CCT-271850, TAE-684, LEE-011, and palbociclib (*P* < 0.001). The higher FAM3D expression, the stronger drug sensitivity of elesclomol, GSK-1904529A, linsitinib (*P* < 0.001). Meanwhile, the higher FAM3D expression, the weaker drug sensitivity of PI-103 (*P* < 0.001).

### Comprehensive results of prognosis and functional enrichment analysis of FAM3C in pancreatic carcinoma

**Figure 7:**
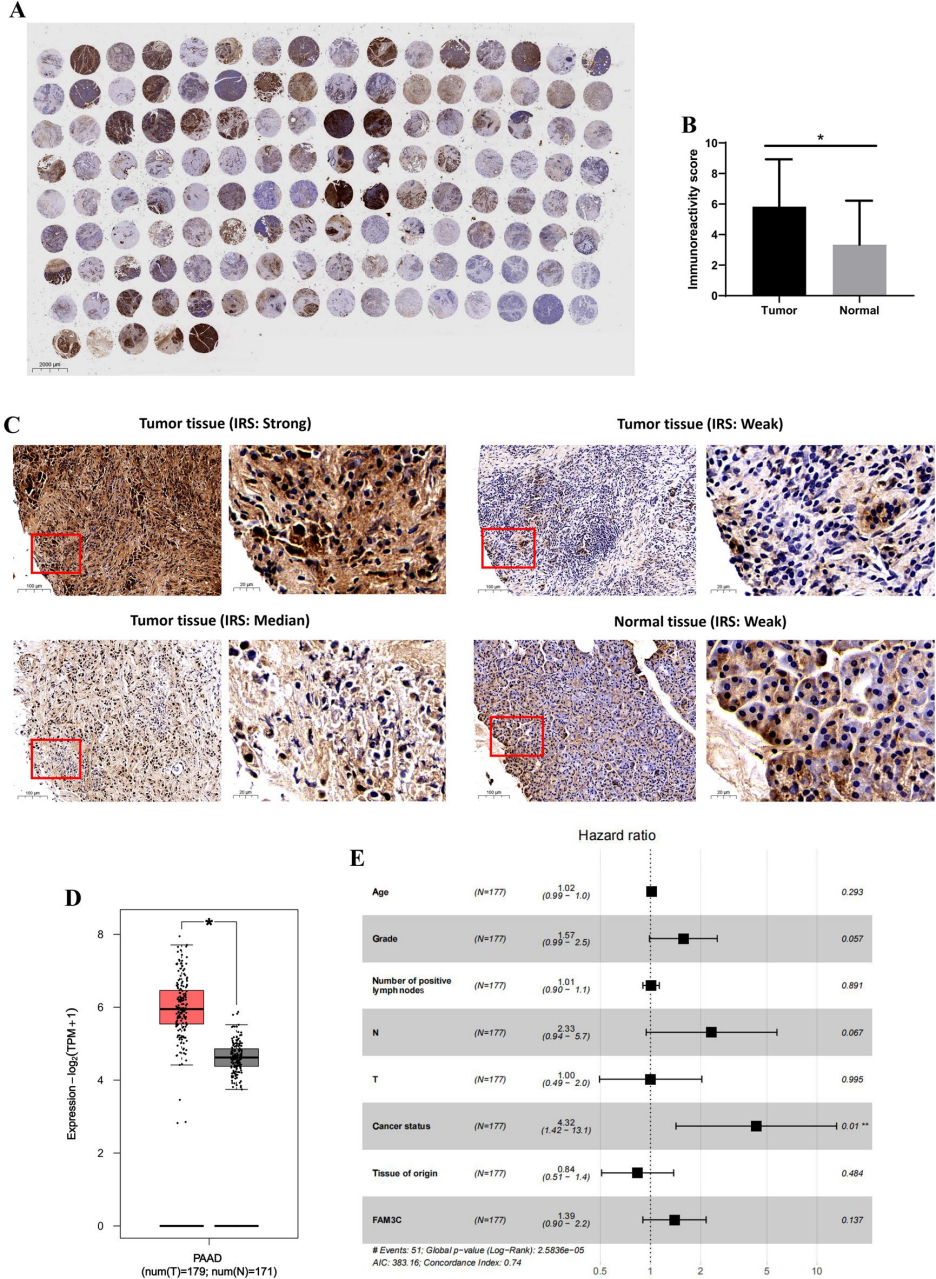

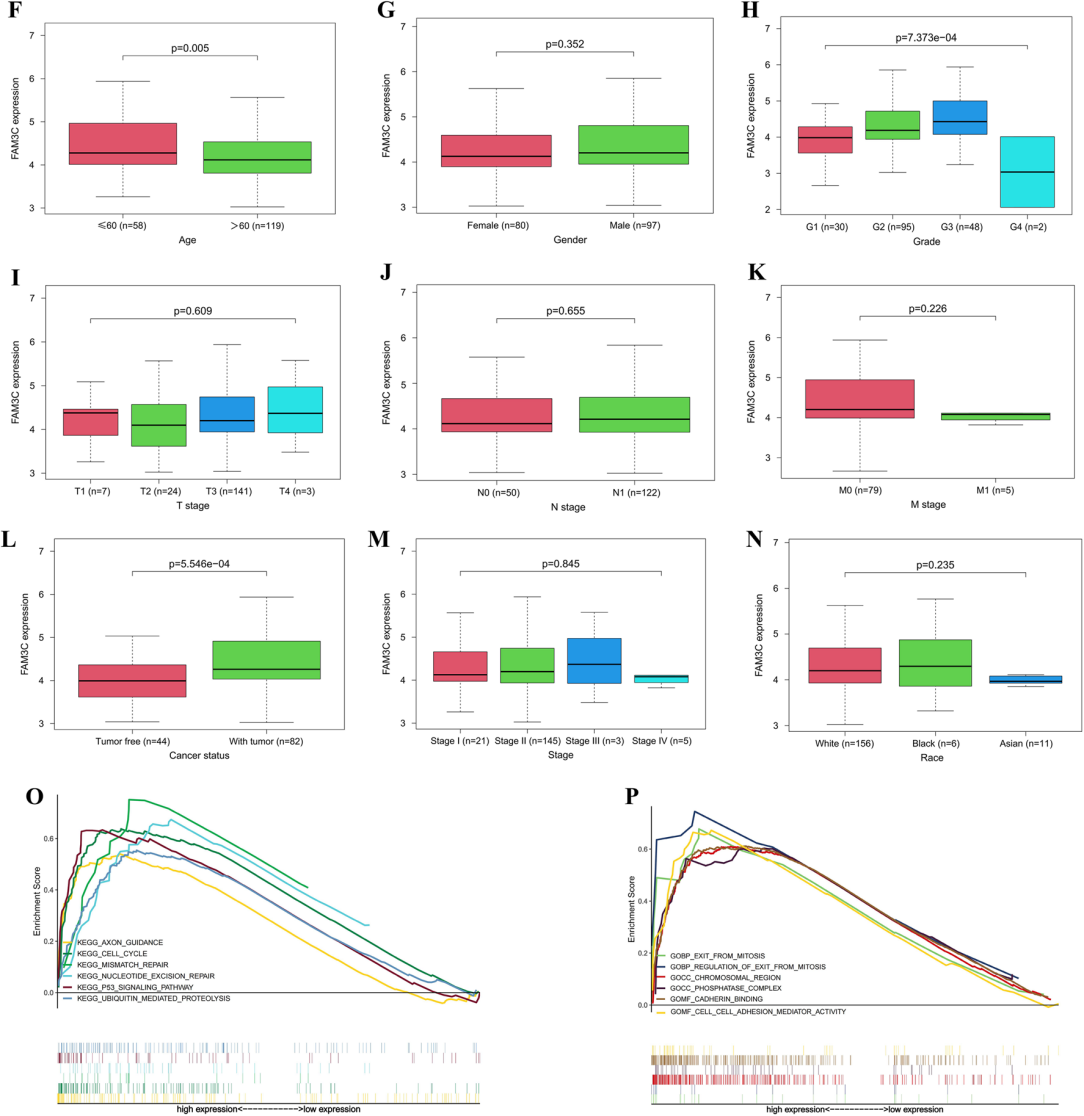
Integrated analysis results of FAM3C in pancreatic carcinoma based on clinical samples, TCGA and GTEx databases. (**A**) A full view of the immunohistochemistry staining of FAM3C in the TMA cohort with 115 pancreatic carcinoma tissues and 18 normal tissues. (**B**) The IRS value of FAM3C protein in pancreatic carcinoma tissues was significantly higher than that in normal tissues. *P < 0.05. (**C**) Representative images of FAM3C immunostaining on the pancreatic carcinoma tissues and normal tissues microarray (original magnifications × 100 and × 400). (**D**) The expression of FAM3C in tumor tissue was significantly higher than normal. *P < 0.05. (**E**) Multivariate analysis results of the cox proportional hazards model. (**F–N**) The results of clinical characterization of FAM3C expression and age (*P* = 0.005) (**F**), gender (*P* = 0.352) (**G**), grade (*P* < 0.001) (**H**), T stage (*P* = 0.609) (**I**), N stage (*P* = 0.655) (**J**), M stage (*P* = 0.226) (**K**), cancer status (*P* < 0.001) (**L**), stage (*P* = 0.845) (**M**), race (*P* = 0.235) (**N**). (**O**) GO enrichment plots from GSEA. Top 2 pathways enriched in the BP, CC, MF. (**P**) KEGG enrichment plots from GSEA. Top 6 pathways enriched in the KEGG.

In order to comprehensive exploration, we combined data from the clinical samples, TCGA and GTEx databases to analyze FAM3C expression in tumor and normal tissues of pancreatic carcinoma. Figure 7A and C showed the immunohistochemistry staining results of FAM3C in the clinical cohort. The expression of FAM3C in tumor tissue was significantly higher than normal (Fig. 7B,D; *P* < 0.05). The results of Cox univariate and multivariate regression analysis of the correlation of FAM3C expression with overall survival among pancreatic as Fig. 7E and Supplementary Table 2. The univariate analysis suggested that age, grade, T stage, N stage, number of positive lymph nodes, cancer status, tissue of origin, and FAM3C expression were risk factors for pancreatic cancer patients prognosis. Multivariate analysis with the cox proportional hazards model indicated that cancer status was independent risk factor for pancreatic cancer patients. Figure 7F–N showed that FAM3C expression associated significantly with grade, age (60 years old as the boundary), and cancer status (*P* < 0.05), but not with gender, T stage, N stage, M stage, stage, and race (*P* > 0.05). From the analysis results of the in-house data, we found that the more serious duodenal invasion, worse differentiation and liver metastasis, the higher expression of FAM3C protein (Table 1). The results of GSEA included GO (BPs (biological process), CCs (cellular components), MFs (molecular functions)) and KEGG^32–34^ enrichment results. We obtained the among the top few enrichment results of high phenotype group based on NES as shown in Fig. 7O,P and Supplementary Table 3. For KEGG, we observed that possible signaling pathways of FAM3C in pancreatic carcinoma were axon guidance, cell cycle, mismatch repair, nucleotide excision repair, p53 signaling pathway, and ubiquitin mediated proteolysis. Furthermore, BP, CC, and MF analysis showed FAM3C was correlated with exit from mitosis, regulation of exit from mitosis, chromosomal region, phosphatase complex, cadherin binding, and adhesion mediator activity between cells.

### FAM3C promoted invasion and metastasis of SW1990 cells

**Figure 8:**
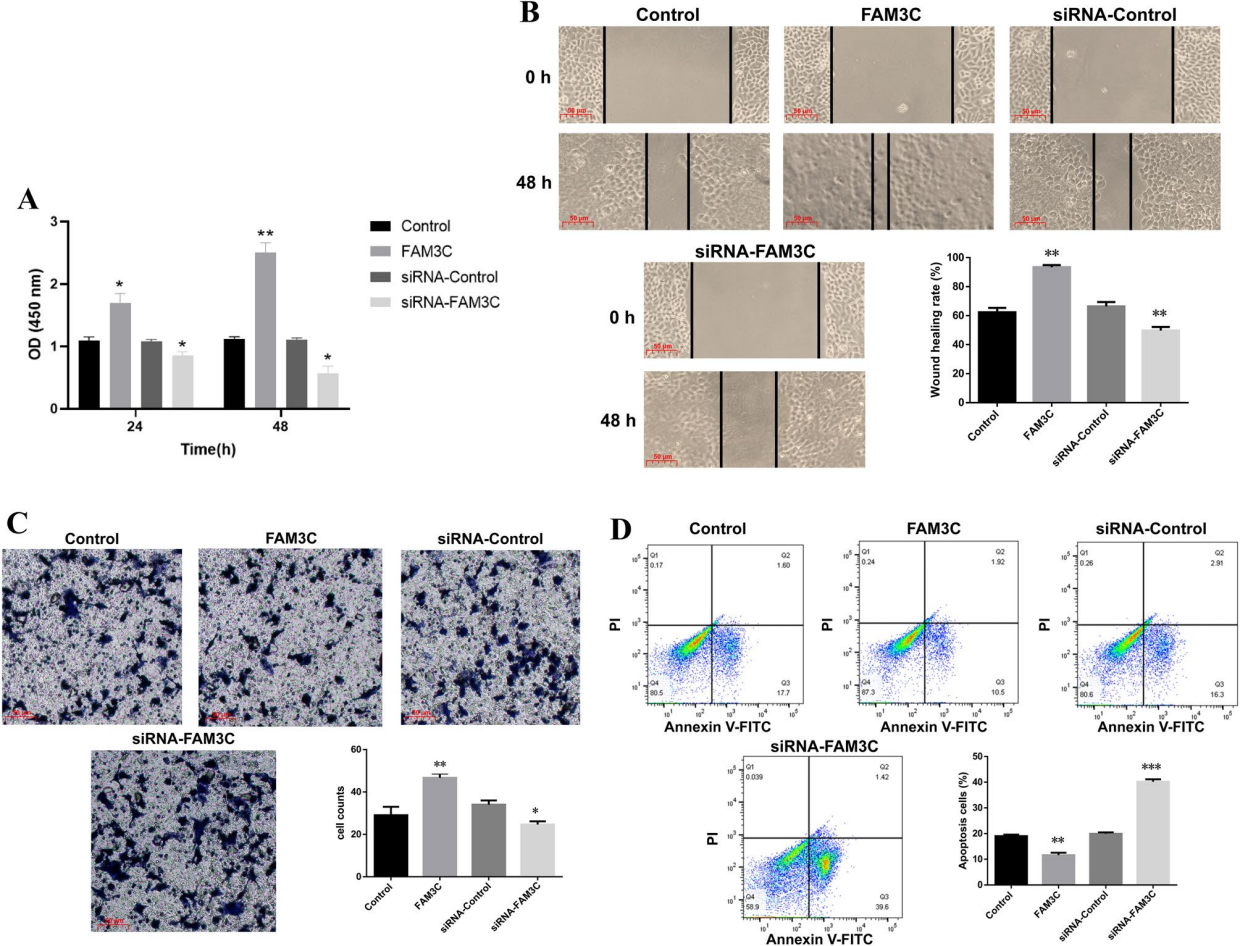
Cell proliferation, migration, invasion and apoptosis in SW1990 cells. (**A**) The effect of FAM3C on the growth of SW1990 cells was analyzed by CCK8 assay. (**B**) Overexpression FAM3C promoted SW1990 cell migration, and knockdown of FAM3C suppressed SW1990 cell migration using wound-healing assay. The magnification of the scratch graph is × 200. (**C**) Overexpression FAM3C promoted SW1990 cell invasion, and knockdown of FAM3C suppressed SW1990 cell invasion using Transwell invasion assay. The magnification of the invasion graph after 24 h is × 200. (**D**) FAM3C had significant influence on the apoptosis of SW1990 cell lines. *P < 0.05; **P < 0.01; ***P < 0.001.

Cell proliferation, migration, invasion and apoptosis are crucial to tumor occurrence and progression. So, we further investigated the effect of FAM3C expression level on tumor cellular functions. As Fig. 8A showed that the OD value of siRNA-FAM3C group all smaller than siRNA-Control at 24 h and 48 h (*P* < 0.05). The wound-healing assay results uncovered that the wound healing rate of siRNA-FAM3C group lower than siRNA-Control (*P* < 0.01) (Fig. 8B). The results of transwell assay showed that the cells counts of lower chamber of siRNA-FAM3C group fewer than siRNA-Control (*P* < 0.05) (Fig. 8C). These results demonstrated that knockdown of FAM3C suppressed SW1990 cells proliferation, migration, and invasion. Meanwhile, The results of apoptosis assay showed that in comparison with siRNA-Control group, siRNA-FAM3C group significantly promoted the apoptosis of SW1990 cells (*P* < 0.001) (Fig. 8D). On the contrary, overexpression FAM3C promoted SW1990 cell proliferation, migration, and invasion while suppressed their apoptosis (Fig. 8A–D).

## Discussion

The protein sequences of each member of FAM3 family genes contain 224–235 amino acids, and the protein sequences homology comparison among each member of FAM3 family genes are from 28.0 to 53.3%^14^. In recent years, with the advancement of detection technology, the study of disease relatedness at the molecular level has been intensified, and the etiology of diseases has been gradually explored in depth, providing a large number of clues for the prevention and treatment of human diseases as a whole. So far, studies have shown that FAM3A can regulate vascular endothelial cell formation and chondrocyte regulation, inhibit hepatic gluconeogenesis and adipogenesis, and protect neuronal morphology and cognitive function^35–37^. FAM3B, also known as PANcreatic-DERived factor (PANDER), is subcellularly localized in secretory vesicles within β cells, regulates blood glucose levels through interactions with the endocrine pancreas and liver^38^. Currently, FAM3B, FAM3C and FAM3D have been shown to be closely associated with the development of solid tumors in humans^22,25,27^. Nevertheless, studies on the comprehensive role of FAM3 family genes in pan-cancer have not been reported. In this study, our aim was to explore in depth the role of FAM3 family genes in pan-cancer based on a comprehensive analysis of public databases. We were able to identify some interesting findings for each individual gene and individual tumor.

In our study, we first explored the expression and prognosis of FAM3 family genes in 33 human cancers. The results demonstrated FAM3A was highly expressed in LICH tumor tissues compared to their normal counterparts, and high expression of FAM3A was significantly associated with poor prognosis in LICH patients. Overexpression of FAM3D-AS1 inhibits the proliferation and invasion of colon cancer cells^39^. FAM3D-AS1 reverses the EMT process and inhibits the development of colorectal cancer through the NF-kB signaling pathway^39^. Likewise, our study showed that the expression of FAM3D was significantly higher in normal colorectal tissues than in tumor tissues. In addition, our study also revealed that FAM3D was lowly expressed in BRCA, HNSC tumor tissues, and low FAM3D expression was negatively correlated with poor prognosis, implying that FAM3D exhibits anti-oncogene properties in BRCA and HNSC. Our results showed that FAM3C was highly expressed in HNSC tumor tissues and high expression of FAM3C was strongly associated with poor prognosis. The expression of FAM3C was significantly higher in oral squamous carcinoma tissues than in normal mucosa, and high expression of FAM3C is strongly associated with worse prognosis^40^. Furthermore, the expression of FAM3C mRNA in esophageal cancer tissues is significantly higher than that in normal tissues, and the high FAM3C expression group have worse prognosis^41^. FAM3C in the inhibited state inhibits the proliferation and invasion of esophageal cancer^42^. These studies coincided with our findings. The results of heatmap revealed that FAM3A and FAM3C were commonly highly expressed in pan-cancer tissues. Meanwhile, among the FAM3 family genes FAM3C had the highest expression in pan-cancer tissues. Previous studies have shown that FAM3C plays an important role in the development of multiple tumors in humans^25^. However, the effect of FAM3C on PAAD has not been reported. In the results of the survival analysis in this study, it was seen that the prognosis of PAAD patients in the high FAM3C expression group was significantly worse compared to the low expression group. Therefore, we further investigated the significance of FAM3C in PAAD.

Thorsson et al. find C4 and C6 subtypes exhibit a composite sign of predominantly macrophages, low lymphocyte infiltration, and high M2 macrophage content, consistent with an immunosuppressed TME for which predict the worst prognosis. Conversely, C2 and C3 belong to two subtypes of type I immune response predicting the best prognosis for tumors^13^. Our study showed the highest expression of FAM3C in the C6 subtype in pan-cancer, predicting that high FAM3C expression was associated with poor prognosis in tumors, consistent with studies suggesting FAM3C is highly expressed in human solid tumors^25^. FAM3D expression was significantly higher in C2 and C3 subtypes than in C4 and C6 subtypes in pan-cancer and BRCA, which implied that the higher FAM3D expression in tumor tissues the better prognosis of patients. This was in line with the anti-oncogene properties of FAM3D in BRCA. FAM3C in LAUD and LUSC had the highest expression in C4 and C6 subtypes conferred the worse prognosis. ILEI facilitates epithelial–mesenchymal transition (EMT) and subsequent metastatic progression of lung cancer^43^. Soldevilla et al. find there are only 5 immune subtypes in colorectal cancer except C5^44^. Our study also showed the same results. Furthermore, we also found that the expression of FAM3D in colorectal cancer was generally higher in all immune subtypes compared to other members of the FAM3 family gene, and the highest expression of FAM3D was the C3 subtype. FAM3D has been verified to inhibit the proliferation, invasion and EMT process of colorectal cancer cells^39^. Immune subtypes could help us understand the prognostic relationship between FAM3 family genes and pan-cancer.

The TME consists of immune cells and stromal components, and whatever the component is, it plays an crucial part in the development, progression and chemoresistance of malignant tumors^45^. The scores of estimate, stromal and immune cells are negatively correlated with the tumor purity^46,47^. In our study, FAM3A expression was negatively correlated with estimate score, stromal score and immune score in most cancer species, which meant that higher FAM3A expression was associated with lower TME content and higher tumor purity. One of the most recognized barriers to anti-tumor immunotherapy is the immunosuppression created by the TME^48^. Cancer cells modify their therapeutic response during treatment by altering their interaction with the host TME^45^. Synergistic hindrance of drug distribution by TME components is a prerequisite for the efficacy of nanoparticles and small molecule drugs^49^. When the TME content were lower than tumor purity, chemotherapeutic drugs may more easily infiltrate cancer cells and exert better therapeutic effects. This inference required further study to explore its accuracy. RNAss and DNAss scores range from 0 to 1, with 0 indicating highly differentiated and 1 indicating undifferentiated^50,51^. In this study, we interestingly found that the lower the RNAss and DNAss scores of TGCT and DLBC when the expression of FAM3 family genes were higher, the fewer stem cell features of the tumors and the higher differentiation. In addition, we also found that FAM3A expression in PAAD was positively correlated with stem cell scores (RNAss and DNAss). FAM3A, FAM3B and FAM3D expression in PAAD were negatively correlated with TME scores, providing ideas for the immune response research of PAAD.

CellMiner Cross-Database (CellMinerCDB) database is directly accessible through a web interface which focuses on analyzing cancer patient-derived human cell line molecular and pharmacological data^52^. Recent studies have focused on using CellMinerCDB to reveal the relationship between cancer drug sensitivity and gene expression, genomic alterations and cell line subgroups^53–55^. In the present study, we found that FAM3 family genes expression were positively correlated with the sensitivity of most anticancer drugs. For instance, FAM3B expression was positively correlated with barasertib drug sensitivity. The higher the expression of FAM3B, the higher the sensitivity of barasertib drug and the better the anticancer effect. Barasertib has been suggested to have therapeutic potential in leukemia, lymphoma, gastric cancer, lung cancer, and breast cancer^56^. The practical significance of the interaction between FAM3C expression and Barasertib drug sensitivity needed to be further experimentally verified. Meanwhile, FAM3D expression was negatively correlated with PI-103 drug sensitivity. PI-103 is a DNA-PK/PI3K/mTOR inhibitor that has been used in preclinical studies, and demonstrated anti-proliferative effects on a variety of cancer types^57,58^. To sum up, the results of drug sensitivity analysis could help us understand the associations between FAM3 family genes expression and numerous anticancer drugs, which provided some ideas for exploring tumor therapy and avoiding tumor drug resistance.

Our study showed that the expression of FAM3C in pancreatic carcinoma tissues was significantly higher than normal tissues, and the high expression of FAM3C was significantly related to poor prognosis of pancreatic carcinoma patients. In vitro cell function experiments showed that FAM3C promoted the proliferation, invasion and migration of SW1990 cells. Previous studies reveal that the expression of FAM3C in human colorectal and gastric cancer tissues is significantly higher than in matched adjacent normal tissues, and FAM3C overexpression is significantly associated with the depth of tumor invasion, lymph node metastasis and TNM stage^59,60^. Moreover, in human breast cancer tissues, FAM3C protein expressions are increased, and FAM3C activates YY1-HSF1 signalling axis to promote the proliferation and migration of breast cancer MDA-MB-231 and BT-549 cells^61^. In the gastric cancer cell lines MKN45 and AGS, knockdown of FAM3C reduces cell migration, and suppresses activation of the PI3K-Akt signaling pathway^62^. MiR-574-3p represses the proliferation and invasion of esophageal cancer by regulating FAM3C and MAPK1^63^. As these studies show that FAM3C promotes the formation of tumors, and exhibits the characteristics of oncogenes. Our study also showed that FAM3C facilitated the progression of PAAD. Chen et al. shows that the results of GSEA analysis could provide some guidance for the discovery of new functions and pathways^63^. We compared GSEA results between low and high FAM3C expression data sets. The results showed that the up-regulated FAM3C was mainly enriched in p53 signaling pathway. Notably, p53 is located on chromosome 17p and regulates DNA repair, cell apoptosis, cell cycle arrest or senescence, thereby exerting its role in tumor suppression^64,65^. FAM3C may be involved in the regulation of PAAD through the p53 signaling pathway. FAM3C may be a new biomarker of PAAD, providing new ideas for the treatment of PAAD.

## Conclusions

In summary, our study observations had demonstrated that the expression of FAM3 family genes was significantly increased in the tumor tissues of pan-cancer and was associated with clinical prognosis. Meanwhile, FAM3 family genes played a crucial role in the immune microenvironment of pan-cancer. Our study also identified that FAM3C was involved in the occurrence and progression of PAAD.

### Supplementary Information

Supplementary Information.

## Data Availability

Transcription expression (RNA-sep—FPKM), phenotype and survival data of 33 human malignant tumors were download from GDC TCGA of UCSC Xena (https://xenabrowser.net/datapages/). Immune subtype, RNA based (RNA-exp) and DNA methylation based (DNA-meth) stemness scores data were download from TCGA Pan-Cancer of UCSC Xena. DTP NCI-60 average z scores and RNA-exp composite expression data were download from CellMiner (https://discover.nci.nih.gov/cellminer/loadDownload.do).
