# Supplementary material for: FAM3 family genes are associated with prognostic value of human cancer: a pan-cancer analysis
Source: Sci Rep. 2023 Sep 13;13:15144. doi: 10.1038/s41598-023-42060-x (PMC10499837; doi:10.1038/s41598-023-42060-x)
Supplement: Supplementary file 1 — Supplementary Information. [file 41598_2023_42060_MOESM1_ESM.pdf]

Supplementary Figure 1. Survival analysis of FAM3 family genes in pan-cancer. The other 8 analysis results. **A-C** Survival curve of FAM3B in MESO **A**, PCPG **B**, SKCM **C**. **D-F** Survival curve of FAM3C in ESCA **D**, GBM **E**, SKCM **F**. **G-H** Survival curve of FAM3D in DLBC **G**, THCA **H**.

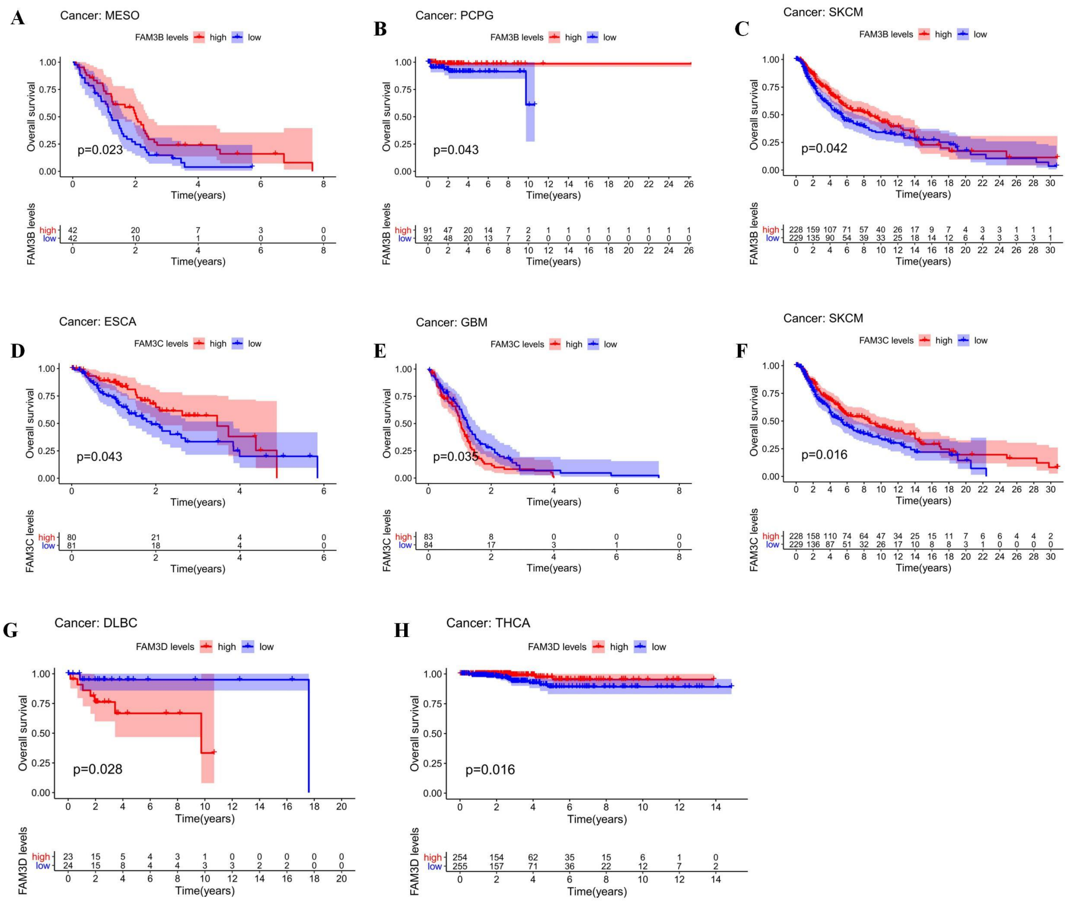

Supplementary Table 1. Full results of the correlation analysis of FAM3 family genes expression and drug sensitivity in CellMiner.

Gene	Drug name	Cor	*p*-value
FAM3B	Barasertib	0.651	0.000
FAM3D	Elesclomol	0.561	0.000
FAM3D	GSK-1904529A	0.559	0.000
FAM3D	Linsitinib	0.535	0.000
FAM3B	AMG-900	0.526	0.000
FAM3B	LDK-378	0.516	0.000
FAM3B	PF-06873600	0.505	0.000
FAM3B	Dexrazoxane	0.480	0.000
FAM3D	PI-103	-0.476	0.000
FAM3B	AM-5992	0.467	0.000
FAM3B	CFI-400945	0.453	0.000
FAM3B	CG-806	0.443	0.000
FAM3B	CCT-271850	0.443	0.000

FAM3B	TAE-684	0.439	0.001
FAM3B	LEE-011	0.435	0.001
FAM3B	Palbociclib	0.431	0.001
FAM3B	NTRC-0066-0	0.428	0.001
FAM3B	CEP-37440	0.417	0.001
FAM3B	CT-GSK183	0.416	0.001
FAM3B	GSK-1070916	0.413	0.001
FAM3B	R-547	0.413	0.001
FAM3B	CEP-14083	0.407	0.001
FAM3D	BAY-876	0.407	0.001
FAM3D	AEW-541	0.406	0.001
FAM3B	CX-5461	0.404	0.002
FAM3B	BAY-1161909	0.403	0.002
FAM3D	BMS-754807	0.402	0.002
FAM3D	ADW-742	0.401	0.002
FAM3B	AZD-3463	0.401	0.002
FAM3A	Rapamycin	0.398	0.002
FAM3B	CC-671	0.397	0.002
FAM3B	SOMCL-12-81	0.395	0.002
FAM3A	SNS-314	0.392	0.002
FAM3C	CC-90003	0.39	0.002
FAM3C	ARRY-704	0.386	0.003
FAM3B	BOS-172722	0.385	0.003
FAM3C	Cobimetinib (isomer 1)	0.385	0.003
FAM3A	Olaparib	0.382	0.003
FAM3A	XL-147	0.379	0.003
FAM3D	Ribavirin	0.378	0.003
FAM3C	RO-4987655	0.377	0.003
FAM3A	Sabutoclax	0.375	0.003
FAM3C	TAK-733	0.374	0.004
FAM3A	LGK-974	0.373	0.004
FAM3A	Quizartinib	0.371	0.004
FAM3D	PLX-8394	0.369	0.004
FAM3C	PD-0325901	0.367	0.004
FAM3C	LXH-254	0.367	0.004
FAM3B	TAS-116	0.367	0.004
FAM3B	ensartinib	0.365	0.005
FAM3C	CCT-251545	-0.364	0.005
FAM3D	AZD-3463	0.360	0.005
FAM3C	AZ-628	0.360	0.005
FAM3C	AZD-0364	0.359	0.005
FAM3C	Acetalax	-0.358	0.005
FAM3A	RAPAMYCIN	0.358	0.005
FAM3B	P-529	-0.355	0.006

FAM3B	ABT-348	0.355	0.006
FAM3D	BMS-536924	0.354	0.006
FAM3C	Pimasertib	0.354	0.006
FAM3B	TAE-226	0.353	0.006
FAM3D	P-529	-0.352	0.006
FAM3C	Simvastatin	0.352	0.006
FAM3C	DIGOXIN	-0.352	0.006
FAM3B	Telatinib	-0.351	0.006
FAM3A	Itraconazole	0.349	0.007
FAM3B	Alisertib	0.349	0.007
FAM3A	AZD-5363	0.347	0.007
FAM3B	PYRAZOLOACRIDINE	0.346	0.007
FAM3B	CCT-251545	0.345	0.007
FAM3C	SCH-772984	0.345	0.008
FAM3C	ARRY-162	0.344	0.008
FAM3B	Danusertib	0.344	0.008
FAM3C	Refametinib	0.343	0.008
FAM3A	FH-535	0.341	0.008
FAM3B	Volasertib	0.340	0.009
FAM3B	BX-912	0.339	0.009
FAM3B	VX-689	0.339	0.009
FAM3C	AMD-070	0.338	0.009
FAM3A	Cpd-401	0.336	0.009
FAM3B	Ifosfamide	0.332	0.010
FAM3C	SEL-120	0.332	0.010
FAM3B	PX-316	0.332	0.010
FAM3A	Irinotecan	0.331	0.010
FAM3A	Axitinib	0.330	0.011
FAM3B	LY-3154567	0.330	0.011
FAM3D	CT-GSK183	0.329	0.011
FAM3C	FGF-401	0.329	0.011
FAM3D	Barasertib	0.328	0.011
FAM3C	RO-5126766	0.327	0.011
FAM3D	Cisplatin	-0.327	0.012
FAM3B	BAY-1217389	0.326	0.012
FAM3B	GSK-1904529A	0.326	0.012
FAM3B	TAK-901	0.323	0.013
FAM3C	AZ-505	0.323	0.013
FAM3A	ARRY-704	-0.322	0.013
FAM3D	SR16157	0.320	0.013
FAM3C	PD 184352	0.320	0.013
FAM3D	TAE-684	0.320	0.014
FAM3C	Procarbazine	0.319	0.014
FAM3C	Kahalide F	0.319	0.014

FAM3A	Temsirolimus	0.319	0.014
FAM3B	EXEL-2280	0.319	0.014
FAM3B	EMD-534085	0.318	0.014
FAM3B	By-Product of CUDC-305	0.317	0.014
FAM3C	Trametinib	0.317	0.014
FAM3A	EMD-1204831	0.315	0.015
FAM3B	KW-2449	0.313	0.016
FAM3B	AT-9283	0.313	0.016
FAM3B	PF-562271	0.313	0.016
FAM3D	Lapachone	-0.312	0.016
FAM3D	DOLASTATIN 10	0.311	0.017
FAM3B	KPT-9274	0.310	0.017
FAM3A	Bosutinib	-0.310	0.017
FAM3D	Vemurafenib	0.309	0.017
FAM3B	Crizotinib	0.308	0.017
FAM3A	pyridoclax	0.308	0.018
FAM3A	Ipatasertib	0.308	0.018
FAM3C	Fluorouracil	-0.308	0.018
FAM3C	ulixertinib	0.307	0.018
FAM3A	5-Fluoro deoxy uridine 10mer	0.306	0.018
FAM3C	CEP-40783	0.306	0.018
FAM3A	Hydrastinine HCl	0.306	0.018
FAM3C	JNJ-38877605	0.305	0.019
FAM3B	AMG-458	-0.304	0.019
FAM3C	RG-7602	-0.304	0.019
FAM3B	AT-13387	0.304	0.019
FAM3A	7-Ethyl-10-hydroxycamptothecin	0.304	0.019
FAM3B	A-911	0.304	0.019
FAM3B	Elesclomol	0.303	0.020
FAM3B	TP-0903	0.303	0.020
FAM3D	PLX-4720	0.302	0.020
FAM3B	Tamoxifen	0.302	0.020
FAM3B	VX-944	0.301	0.021
FAM3D	GDC-0994	0.301	0.021
FAM3A	Triethylenemelamine	0.300	0.021
FAM3A	Amuvatinib	0.299	0.021
FAM3D	Dabrafenib	0.299	0.021
FAM3D	IDH-C227	-0.299	0.021
FAM3A	LY-294002	0.299	0.022
FAM3C	RG-6016	0.298	0.022
FAM3C	Milciclib	0.297	0.022
FAM3A	Cisplatin	0.297	0.022
FAM3D	AM-5992	0.297	0.022
FAM3A	AZD-3514	0.297	0.023

FAM3A	Uracil mustard	0.296	0.023
FAM3D	BGB-283	0.295	0.023
FAM3B	PF-06463922	0.295	0.023
FAM3A	Avagacestat	0.295	0.023
FAM3A	Etoposide	0.295	0.024
FAM3D	OTS-964	-0.294	0.024
FAM3B	JNJ-47117096	0.293	0.024
FAM3A	enantiomer of PF-4176340	0.293	0.024
FAM3A	Topotecan	0.292	0.025
FAM3B	brigatinib	0.292	0.025
FAM3C	EPZ-020411	0.292	0.025
FAM3B	KPT-8602	0.291	0.025
FAM3C	Bisacodyl, active ingredient of Viraplex	-0.290	0.026
FAM3A	TAK-733	-0.290	0.026
FAM3A	Chlorambucil	0.289	0.026
FAM3B	Tanespimycin	0.289	0.027
FAM3D	Quercetin	-0.288	0.027
FAM3A	JZL-195	0.287	0.027
FAM3D	CEP-37440	0.287	0.027
FAM3B	Erlotinib	-0.287	0.027
FAM3C	Axitinib	-0.287	0.028
FAM3B	XAV-939	-0.287	0.028
FAM3C	Pelitrexol isomer A	-0.287	0.028
FAM3A	(+)-JQ1	0.286	0.028
FAM3C	P-529	0.286	0.028
FAM3B	SR16157	0.286	0.028
FAM3D	LDK-378	0.285	0.028
FAM3C	By-Product of CUDC-305	-0.285	0.028
FAM3A	BAY-87-2243	0.285	0.029
FAM3B	GNE-617	0.284	0.029
FAM3C	LY-3009120	0.284	0.030
FAM3C	LY-3214996	0.283	0.030
FAM3A	Elliptinium Acetate	0.283	0.030
FAM3A	INCB-047775	0.282	0.030
FAM3A	Teniposide	0.282	0.031
FAM3A	SAR-245409	0.280	0.031
FAM3D	TAE-226	0.280	0.032
FAM3D	Palbociclib	0.280	0.032
FAM3B	BMS-754807	0.280	0.032
FAM3B	Belinostat	0.279	0.032
FAM3A	PD-0325901	-0.279	0.032
FAM3B	YK-4-279	0.279	0.033
FAM3A	LY-3214996	-0.279	0.033
FAM3A	Cobimetinib (isomer 1)	-0.278	0.033

FAM3A	Mitomycin	0.278	0.033
FAM3A	S-49076	0.278	0.033
FAM3B	benzaldehyde (BEN)	0.278	0.033
FAM3A	Trametinib	-0.278	0.033
FAM3B	PI-103	-0.278	0.033
FAM3A	Quercetin	0.278	0.033
FAM3C	SGX-523	0.277	0.034
FAM3A	By-Product of CUDC-305	-0.277	0.034
FAM3B	ARQ-621	0.277	0.034
FAM3A	Thiotepa	0.276	0.034
FAM3A	Pipobroman	0.276	0.034
FAM3C	XAV-939	0.276	0.034
FAM3C	JNJ-54302833	-0.275	0.035
FAM3A	BMN-673	0.272	0.037
FAM3A	Calusterone	0.272	0.037
FAM3B	Teglarinad	0.272	0.037
FAM3A	E-7820	-0.272	0.037
FAM3B	CEP-28122	0.270	0.038
FAM3A	OSI-027	0.269	0.040
FAM3C	benzaldehyde (BEN)	-0.269	0.040
FAM3B	TAK Plk inhibitor	0.268	0.040
FAM3B	ADW-742	0.268	0.040
FAM3D	ST-3595	-0.267	0.041
FAM3B	MLN-0905	0.267	0.041
FAM3B	XR-11576	0.267	0.041
FAM3B	Linsitinib	0.267	0.041
FAM3A	Floxuridine	0.267	0.041
FAM3A	Deforolimius	0.267	0.041
FAM3C	Selumetinib	0.266	0.042
FAM3B	XL-147	-0.266	0.042
FAM3B	PF-03758309	0.265	0.042
FAM3D	AMG-900	0.265	0.042
FAM3A	Mitoxantrone	0.265	0.043
FAM3A	Salinomycin	0.264	0.043
FAM3B	MPC-3100	0.264	0.043
FAM3C	Pelitrexol	-0.264	0.044
FAM3B	Pipamperone	0.264	0.044
FAM3A	Neratinib	-0.263	0.044
FAM3A	WORTMANNIN	0.263	0.044
FAM3A	ZM-336372	0.263	0.044
FAM3A	Melphalan	0.263	0.044
FAM3A	PKM2 (9)	0.262	0.045
FAM3C	CC-671	-0.262	0.045
FAM3C	Encorafenib	0.262	0.045

FAM3A	AZD-3229	0.261	0.046
FAM3B	5-aza-T-dCyd	0.261	0.046
FAM3A	SR16157	0.261	0.046
FAM3A	Everolimus	0.261	0.046
FAM3D	Everolimus	-0.261	0.046
FAM3A	MK-2206	0.260	0.046
FAM3D	Bleomycin	-0.260	0.047
FAM3A	MITOXANTRONE	0.260	0.047
FAM3B	BMS-387032	0.259	0.047
FAM3B	B-7100	0.259	0.048
FAM3D	IDF-11774	0.258	0.048
FAM3A	AMG-176	0.258	0.049
FAM3B	VS-4718	0.258	0.049
FAM3B	MI-503	0.257	0.050

Supplementary Table 2. Univariate and multivariate analysis of the relationship between FAM3C expression and overall survival in PAAD.

Parameters	Univariate analysis			Multivariate analysis		
	HR	95%CI	p value	HR	95%CI	p value
Age	1.029	1.008-1.051	0.008	1.016	0.986-1.047	0.293
Gender (Female/Male)	0.809	0.537-1.219	0.311			
Grade (G1/G2/G3/G4)	1.453	1.091-1.934	0.010	1.575	0.986-2.516	0.057
Stage (I/II/III/IV)	1.213	0.842-1.747	0.299			
T (T1/T2/T3/T4)	1.556	1.003-2.413	0.048	1.002	0.494-2.032	0.995
N (N0/N1)	2.153	1.281-3.617	0.004	2.326	0.942-5.743	0.067
M (M0/M1)	0.756	0.181-3.157	0.701			
Family history of cancer (Yes/No)	0.894	0.520-1.537	0.686			
History of chronic pancreatitis (Yes/No)	0.849	0.406-1.778	0.665			
History of diabetes (Yes/No)	1.079	0.619-1.879	0.789			
Alcohol history (Yes/No)	0.872	0.561-1.355	0.543			
Lymph nodes examined count	0.997	0.975-1.019	0.782	1.008	0.9-1.129	0.891
Number of positive lymph nodes	1.062	1.007-1.121	0.027			

Cancer status (Tumor free/With tumor)	7.666	3.260-18.029	0.000	4.317	1.422-13.101	0.010
Race (White/Black/Asian)	0.902	0.593-1.371	0.630			
Tissue of origin (Head of pancreas/Body of pancreas/Tail of pancreas/Overlapping lesion of pancreas)	0.610	0.395-0.941	0.026	0.837	0.509-1.378	0.484
FAM3C expression	1.761	1.287-2.411	0.000	1.393	0.899-2.158	0.137

Supplementary Table 3. Gene sets enriched in phenotype high.

MSigDB collection	Gene set name	NES	NOM p-value	FDR q-val
C2.cp.kegg.v7.4.symbols.gmt [Curated]	KEGG_P53_SIGNALING_PATHWAY	1.913	0.000	0.043
	KEGG_AXON_GUIDANCE	1.741	0.008	0.137
	KEGG_UBIQUITIN_MEDIATED_PROTEOLYSIS	1.835	0.000	0.057
	KEGG_NUCLEOTIDE_EXCISION_REPAIR	1.970	0.000	0.037
	KEGG_CELL_CYCLE	1.864	0.002	0.048
	KEGG_MISMATCH_REPAIR	1.870	0.000	0.060
C5.all.v7.4.symbols.gmt [Gene ontology]	GOBP_EXIT_FROM_MITOSIS	2.132	0.000	0.023
	GOBP_REGULATION_OF_EXIT_FROM_MITOSIS	2.071	0.000	0.053
	GOCC_CHROMOSOMAL_REGION	1.940	0.002	0.055
	GOCC_PHOSPHATASE_COMPLEX	1.957	0.002	0.063
	GOMF_CADHERIN_BINDING	2.143	0.000	0.037
	GOMF_CELL_CELL_ADHESION_MEDIATOR_ACTIVITY	2.046	0.000	0.063

NES=normalized enrichment score, NOM=nominal, FDR=false discovery rate, NOM p value < 0.05 and FDR q-val <0.25 are considered as significant.
